# Primary large B-cell lymphoma of the patella with early systemic progression: A case report

**DOI:** 10.1016/j.ijscr.2025.111344

**Published:** 2025-04-23

**Authors:** Sarra Ben Rejeb, Majdi Ben Romdhane, Majdi Sghaier, Mehdi Charfi

**Affiliations:** a Pathology department, Security Forces Hospital, Marsa, Tunisia; b Orthopedic department, Security Forces Hospital, Marsa, Tunisia

**Keywords:** Primary bone lymphoma, patella, Diffuse large B-cell lymphoma, DLBCL, Non-GCB, Progression

## Abstract

**Introduction:**

Primary bone lymphoma (PBL) of the patella is an extremely rare condition, with fewer than ten documented cases. We herein described a unique case of primary diffuse large B-cell lymphoma (DLBCL) of the patella, highlighting its diagnostic challenges, therapeutic considerations, and unexpectedly rapid systemic progression, contributing valuable insights to the understanding of this uncommon entity.

**Case presentation:**

A 68-year-old woman with a history of papillary thyroid carcinoma reported six months of worsening right knee pain, swelling, and reduced mobility. Imaging showed a lytic patellar lesion with cortical damage and soft tissue involvement. A biopsy confirmed non-germinal center B-cell-like DLBCL, identified by positive CD45, CD20, PAX5, MUM1, and BCL2 markers, and negative CD10, BCL6, and pan-cytokeratin results. PET/CT and bone marrow biopsy confirmed the disease was localized (stage IE). She received R-CHOP chemotherapy, but six months later, PET/CT revealed widespread progression to lymph nodes, adrenals, bones, breasts, and subcutaneous tissues. A breast biopsy matched the original DLBCL. Salvage chemotherapy with the R-ICE regimen (rituximab, ifosfamide, carboplatin, etoposide) was initiated. However, the patient’s clinical condition rapidly deteriorated, and she opted for palliative care.

**Clinical discussion:**

Patellar PBL is challenging to diagnose due to its vague symptoms and similarity to metastases or other bone tumors, particularly in patients with prior cancer. Biopsy and immunohistochemistry were essential for confirmation. The rapid systemic spread, potentially tied to the non-GCB subtype, indicates an aggressive disease course, uncommon for early-stage PBL.

**Conclusion:**

This case contributes to the sparse literature on patellar PBL, stressing the importance of biopsy for diagnosis and the need for close monitoring due to the risk of swift progression, even in localized disease. Further study of prognostic factors is warranted.

## Highlights

• Rare primary bone lymphoma in patella, <10 cases reported, non-GCB DLBCL enriches limited knowledge of this condition.
• Early systemic progression under R-CHOP, aggressive non-GCB subtype suggests unpredictable behavior, challenges treatment.
• Close follow-up critical; rapid progression needs research on prognostic factors, tailored strategies to improve outcomes.

## 1 Introduction

Lymphomas are a heterogeneous group of lymphoid neoplasms primarily arising in lymph nodes [1]. While secondary bone marrow involvement occurs frequently in systemic lymphomas, primary bone lymphoma (PBL)—defined as lymphoma originating in a single bone site (or multiple bone sites) without nodal or extranodal involvement for at least six months post-diagnosis—is rare, accounting for <1 % of primary bone malignancies and approximately 5 % of extranodal lymphomas [1]. Common sites include the femur, pelvis, tibia/fibula, and humerus, whereas patellar involvement is exceedingly uncommon, with fewer than ten reported cases [[2], [3], [4], [5], [6], [7], [8]]. Its rarity and nonspecific clinical and radiological features often lead to misdiagnosis [9]. We herein presented a rare case of primary non-GCB subtype DLBCL of the patella, initially clinically mistaken for metastatic disease due to the patient's oncologic history and the lesion's appearance. This work has been reported in accordance with the SCARE criteria [10].

## 2 Case presentation

**Fig. 1:**
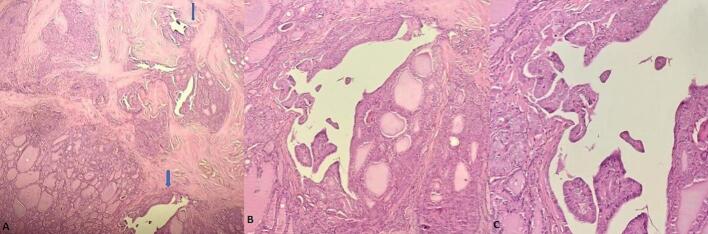
***Pathological features of the thyroid cancer* (A):** Hematoxylin & Eosin, X 4 magnification showing carcinomatous proliferation organized intro papillary and follicular structures set in a fibrous stroma **(B)** Hematoxylin & Eosin x 200: Papillary structures aligned with atypical cells **(C)** Hematoxylin & Eosin x 400:The tumor cells shows typical papillary nuclear atypia with clarification, irregular nuclei and grooves.

**Fig. 2:**
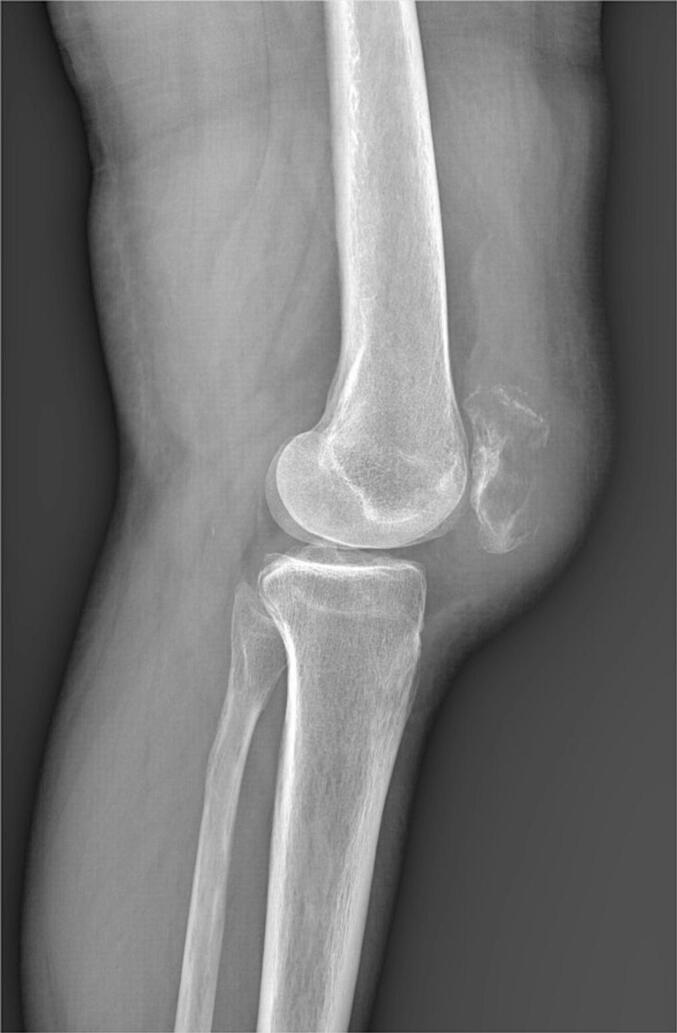
***Conventional radiography:*** Anteroposterior radiograph of the right knee showing a large, poorly defined lytic lesion involving the majority of the patella with cortical thinning.

**Fig. 3:**
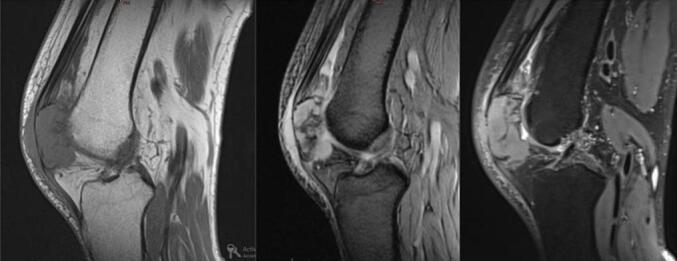
***MRI findings:*** Marrow replacement with low T1 signal intensity, high STIR signal, marked contrast enhancement, cortical disruption, and adjacent soft tissue oedema.

**Fig. 4:**
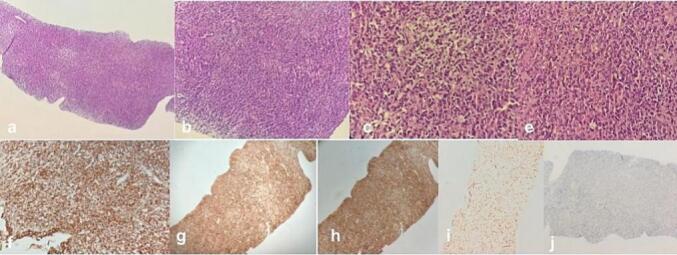
***Pathological Findings* (a-e):** Diffuse tumor proliferation of discohesive tumor cells with scanty cytoplasm and atypical nuclei **(f):** The cells showed strong and diffuse staining for CD20 marker **(g):** The cells showed strong and diffuse staining for CD79 marker **(h):** The cells showed strong and diffuse staining for CD45 marker **(i):** High Ki67 proliferation rate **(j):** The cells showed negative staining for CK.

**Fig. 5:**
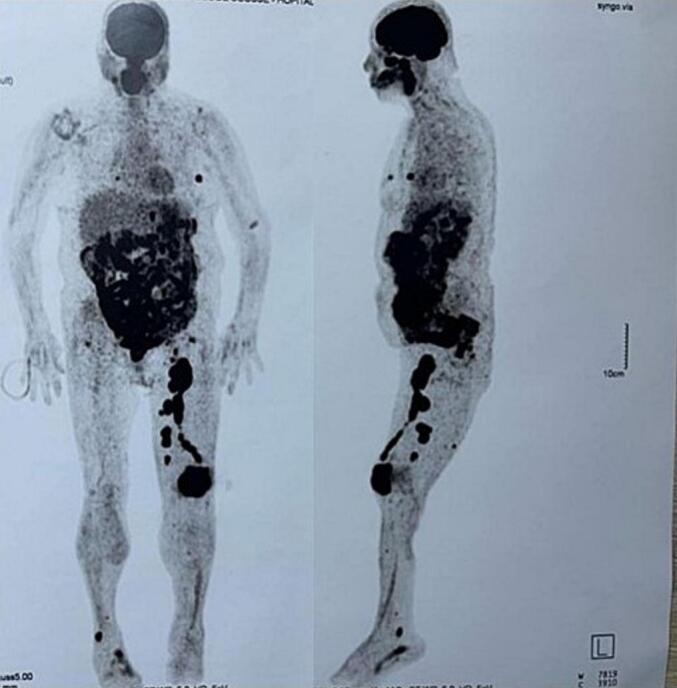
***Follow-up PET/CT demonstrating systemic progression at 6 months:*** Fused FDG PET/CT images showing multiple new sites of intense hypermetabolism including widespread lymphadenopathy bilateral adrenal glands, multiple osseous lesions with persistent marked fixation in the right patella, mammary, and subcutaneous involvement, indicating extensive disease progression.

A 68-year-old Caucasian woman presented with a six-month history of right knee pain, swelling, and reduced range of motion. The pain was described as insidious in onset, progressively worsening, characterized as a persistent, deep, dull ache, rated 6/10 in severity. It was exacerbated by weight-bearing activities like walking and climbing stairs and was partially relieved by rest and over-the-counter analgesics. Nocturnal pain was also present. Her medical history included surgically treated uterine leiomyoma (two years prior) and papillary thyroid carcinoma (diagnosed six years prior, treated with total thyroidectomy and iodine-131 ablation, considered in remission). Review of archived thyroid cancer slides confirmed papillary thyroid carcinoma, with typical nuclear features and architecture; no evidence of lymphoma or lymphoid infiltration was identified ruling out associated lymphoid malignancy (Fig. 1). Physical examination revealed moderate effusion of the right knee, tenderness over the patella, and restricted range of motion (0-90 degrees flexion). There was no palpable lymphadenopathy or organomegaly. The patient reported no constitutional symptoms such as fever, night sweats, or recent unintentional weight loss at initial presentation. Laboratory findings, including lactate dehydrogenase (LDH, 180 U/L; normal range < 250 U/L), complete blood count (Hemoglobin 13.1 g/dL, WBC 7.5 × 10^9^/L, Platelets 280 × 10^9^/L), calcium (9.8 mg/dL), and alkaline phosphatase (ALP, 75 U/L; normal range 40-129 U/L**)** were within normal limits. Radiographs of the right knee demonstrated a large, ill-defined lytic lesion involving almost the entire patella, with cortical thinning and suspected breach (Fig. 2). Computed tomography (CT) confirmed a permeative, destructive lytic lesion with ill-defined margins occupying nearly the entire patella, associated with cortical disruption anteriorly and posteriorly, and subtle soft tissue extension. Magnetic resonance imaging (MRI) showed extensive marrow replacement appearing hypointense on T1-weighted images and hyperintense on STIR sequences, with avid, heterogeneous contrast enhancement. Cortical disruption was clearly visualized along with significant surrounding soft tissue edema and enhancement, extending into the prepatellar bursa and infra-patellar fat pad (Fig. 3). Given the patient's history of thyroid cancer, metastatic disease was initially suspected. A CT-guided core biopsy of the patellar lesion was performed. Histopathology revealed diffuse infiltration by large, atypical lymphoid cells with irregular nuclear contours, vesicular chromatin, prominent nucleoli, and scant cytoplasm, admixed with frequent mitotic figures, set within a fibrous stroma (Fig. 4 A-E). Immunohistochemistry was positive for CD45 (confirming hematopoietic origin), CD20, PAX5 (confirming B-cell lineage), MUM1, and BCL2. The tumor cells were negative for CD138 (ruling out myeloma), CD10, BCL6, C-MYC and pan-cytokeratin (ruling out carcinoma) (Fig. 4 F-J). The Ki-67 proliferation index was high at approximately 70 %. These findings were consistent with a DLBCL, non-GCB subtype based on the Hans algorithm (CD10-, BCL6-, MUM1+). Systemic staging included a bone marrow biopsy, which showed no evidence of lymphoma involvement. A whole-body Fluorodeoxyglucose Positron Emission Tomography/Computed Tomography (FDG PET/CT) scan demonstrated intense, focal hypermetabolic activity confined solely to the right patella (SUVmax 15.2), with no other abnormal FDG uptake in lymph nodes or organs. Based on these findings, the diagnosis was established as primary patellar DLBCL, non-GCB subtype, Ann Arbor stage IE. The patient was treated with the R-CHOP regimen (rituximab, cyclophosphamide, doxorubicin, vincristine, prednisone). The patient completed 6 cycles of R-CHOP chemotherapy. A follow-up PET/CT scan was performed at 6 months and revealed unexpected widespread disease progression with multiple new hypermetabolic lesions involving diffuse lymph nodes (cervical, axillary, mediastinal, abdominal, pelvic), the right adrenal gland, multiple bones (vertebrae, ribs, pelvis), both breasts, and numerous subcutaneous tissues (Fig. 5). A biopsy of one of the hypermetabolic lesions in the left breast was performed and confirmed infiltration by DLBCL with an identical immunohistochemical profile to the original patellar lesion (CD45+, CD20+, PAX5+, MUM1+, BCL2+, CD10-, BCL6-, CK-), confirming systemic progression/relapse of the non-GCB DLBCL.

Salvage chemotherapy with the R-ICE regimen (rituximab, ifosfamide, carboplatin, etoposide) was initiated. However, the patient’s clinical condition rapidly deteriorated, and she opted for palliative care.

## 3 Discussion

Primary bone lymphoma of the patella is exceptionally rare with fewer than ten cases documented in the literature [[1], [2], [3], [4], [5], [6], [7], [8], [9],^11^,^12^]. Given its rarity, it represents a diagnostic and therapeutic challenge [13]. The management and treatment approaches described in these case reports varied widely, resulting in a range of outcomes. While most of the reported cases showed favorable outcomes, our case, presenting as primary patellar non-GCB DLBCL with unexpected rapid systemic progression, adds crucial information to this limited body of knowledge.

The diagnosis of patellar PBL is often challenging because of its nonspecific presentation. Symptoms like localized pain, swelling, and restricted motion, as seen in our patient, mimic more common conditions such as osteoarthritis, infection, metastasis, or other primary bone tumors [3,9,13]. Giant cell tumor of bone (GCTB) is the most frequent primary patellar neoplasm, further complicating the differential [8]. In patients with a history of cancer, such as our patient’s papillary thyroid carcinoma, a metastatic origin should always be considered. However, in this reported case, review of thyroid cancer slides revealed the classic papillary nature of the thyroid cancer without lymphoid elements supporting the primary nature of the patellar lymphoma in this case.

Radiologic findings in patellar PBL, while suggestive of malignancy, lack specificity. As demonstrated in our case, radiographs typically show lytic or mixed lytic-sclerotic lesions, often with ill-defined margins and cortical destruction [3,14]. MRI is highly contributive, showing marrow replacement (typically low T1, high T2/STIR signal), contrast enhancement, and the extent of soft tissue involvement, but these features overlap significantly with metastasis, giant cell tumor, and osteomyelitis [1,14]. FDG PET/CT is the most sensitive imaging modality for detecting lymphoma, showing intense uptake, and is essential for staging by confirming isolated bone involvement (Stage IE) or identifying occult systemic disease (Stage IV) [9,15,16]. In our patient, the initial whole PET/CT confirmed localized disease showing intense, focal hypermetabolic activity confined solely to the right patella with no other hypermetabolic areas especially in lymph nodes. However, the rapid development of widespread systemic disease merely six months later raises questions about the true disease burden at diagnosis. Although the potential for undetected microscopic disease at initial staging is theoretically possible, it clinically seems unlikely. Indeed, PET-scan is the most sensitive imaging modality for staging and response assessment for most lymphomas with high sensitivity (90-95 %) and specificity (85-90 %) due to its ability to detect metabolically active disease in lymph nodes and extranodal sites [17]. While false positives related to hypermetabolic inflammatory lesions are possible, false negatives remain exceptionally rare mainly occurring in low grade lymphomas with lower FDG-avidity [17].

PBL is typically defined as a lymphoma presenting with bone involvement, without evidence of disease in distant lymph nodes, organs, or other sites at the time of diagnosis(for at least 6 months following the initial diagnosis) [9]. In this described case, since the patient developed at 6 months, systemic involvement (that was not clinically or radiologically present at diagnosis), our case still fulfill the initial criteria for PBL. The subsequent spread does not alter the fact that the bone was the primary site. Therefore, we can still retain the primary bone lymphoma origin, acknowledging that the disease has now progressed to involve systemic sites.

The diagnosis of a lymphoma is only made on pathological examination with immunohistochemical analysis. Although, DLBC is the most common lymphoma subtype involving the bones [9], both B-cell and T-cell lymphomas have been reported in the patella [2,3,5,6,13]. In our case, the biopsy revealed features consistent with DLBCL. The immunohistochemical profile was critical not only for diagnosis but also for subtyping and prognosis. Positive staining for CD45 confirmed leukocyte origin, while CD20 and PAX5 established B-cell lineage. Negativity for pan-cytokeratin and CD138 effectively ruled out metastatic carcinoma and plasma cell myeloma, respectively. The pattern of MUM1 positivity alongside negativity for CD10 and BCL6 classified the tumor as the non-germinal center B-cell-like (non-GCB) subtype according to the Hans algorithm [12]. BCL2 positivity was also noted, however as C-MYC was negative, this lymphoma cannot be classified as a double expresser, which is typically associated with aggressive behavior [18]. The non-GCB subtype of DLBCL is generally associated with a less favorable prognosis than the GCB subtype in systemic DLBCL, often driven by constitutive NF-κB pathway activation [19].

The treatment protocol of patellar lymphoma was widely variable. Surgery in PBL is primarily reserved for obtaining diagnostic biopsies or managing pathological fractures. In patellar PBL, complete patellectomy might be considered for extensive destruction, intractable pain, or failure of non-operative treatment, but it carries significant functional consequences, including quadriceps weakness and extensor lag [2]. Therefore, patellar preservation with chemo-radiotherapy is generally preferred. Radiotherapy plays a crucial role in consolidating response and improving local control, especially for bulky disease [9]. Most previously reported patellar PBL cases achieved remission with chemo(radio)therapy, and none described such rapid systemic progression as experienced in this case [[2], [3], [4], [5], [6],^8^,^13^]. A systematic comparison revealed treatments ranging from chemotherapy alone [4], combined modality therapy [3,5], to patellectomy plus chemotherapy [2,7], generally resulting in good initial outcomes.

In this described case, the decision against novel agents initially was based on standard guidelines for localized DLBCL [20]. However, the early systemic progression in our patient highlights potential resistance mechanisms inherent to some non-GCB DLBCLs and raises questions about whether alternative or intensified first-line strategies might be warranted in specific high-risk PBL cases, although evidence for this is currently lacking.

While PBL overall has a relatively good prognosis, with a reported 5-year overall survival rates for localized disease often exceeding 80 %, compared to other primary bone cancers, the prognostic significance of DLBCL subtypes within PBL is less clearly defined, particularly in rare sites like the patella [1,9,21]. Favorable prognostic factors typically include stage IE disease, normal LDH levels, good performance status, and potentially younger age [9,21]. Our case stands out due to the aggressive course despite initially localized (Stage IE) disease. The rapid progression within nearly six months suggests that the non-GCB subtype and possibly the high Ki-67 index (70 %) may have conferred aggressive biology, challenging the typically favorable outlook for Stage IE PBL [19].

The unexpected early systemic progression in this rare case of patellar PBL highlights the need for vigilant monitoring and further research into prognostic factors and accurate management protocols. Future studies should investigate the biological drivers of aggressive behavior in non-GCB DLBCL to optimize treatment strategies.

## 4 Conclusion

This report described an extremely rare case of primary non-GCB DLBCL of the patella. It highlights the diagnostic challenges posed by nonspecific symptoms and radiological findings that mimic more common conditions, emphasizing the key role of timely biopsy and immunohistochemical analysis. Interestingly, this case documented unusually rapid systemic progression despite initial Stage IE disease treated with standard R-CHOP chemotherapy. This aggressive behavior, potentially linked to the non-GCB subtype and high proliferation index, contrasts with previous reports of patellar PBL and the generally favorable prognosis associated with localized PBL. This case contributes significantly to the sparse literature, underscoring the heterogeneity of PBL and the need for vigilant follow-up, particularly in non-GCB subtypes, to detect early progression. It is necessary we continue reporting such cases to better understand prognostic factors and optimize first-line treatment.

## Consent for publication

Written informed consent for publication of patient’s clinical details and clinical images were obtained from the patient.

## Ethics statement

The ethics approval is not required for case reports deemed not.

to constitute research at my institution.

## Sources of funding

None.

## Declaration of competing interest

None.
